# TUCAN/CARDINAL/CARD8 and apoptosis resistance in non-small cell lung cancer cells

**DOI:** 10.1186/1471-2407-6-166

**Published:** 2006-06-23

**Authors:** Agnieszka Checinska, Giuseppe Giaccone, Bas SJ Hoogeland, Carlos G Ferreira, Jose A Rodriguez, Frank AE Kruyt

**Affiliations:** 1 Department of Medical Oncology, VU University Medical Center, 1081 HV Amsterdam, The Netherlands; 2 Department of Clinical and Translational Research, Instituto Nacional de Cancer, 20230-092 Rio De Janeiro, Brazil

## Abstract

**Background:**

Activation of caspase-9 in response to treatment with cytotoxic drugs is inhibited in NSCLC cells, which may contribute to the clinical resistance to chemotherapy shown in this type of tumor. The aim of the present study was to investigate the mechanism of caspase-9 inhibition, with a focus on a possible role of TUCAN as caspase-9 inhibitor and a determinant of chemosensitivity in NSCLC cells.

**Methods:**

Caspase-9 processing and activation were investigated by Western blot and by measuring the cleavage of the fluorogenic substrate LEHD-AFC. Proteins interaction assays, and RNA interference in combination with cell viability and apoptosis assays were used to investigate the involvement of TUCAN in inhibition of caspase-9 and chemosensitivity NSCLC.

**Results:**

Analysis of the components of the caspase-9 activation pathway in a panel of NSCLC and SCLC cells revealed no intrinsic defects. In fact, exogenously added cytochrome c and dATP triggered procaspase-9 cleavage and activation in lung cancer cell lysates, suggesting the presence of an inhibitor. The reported inhibitor of caspase-9, TUCAN, was exclusively expressed in NSCLC cells. However, interactions between TUCAN and procaspase-9 could not be demonstrated by any of the assays used. Furthermore, RNA interference-mediated down-regulation of TUCAN did not restore cisplatin-induced caspase-9 activation or affect cisplatin sensitivity in NSCLC cells.

**Conclusion:**

These results indicate that procaspase-9 is functional and can undergo activation and full processing in lung cancer cell extracts in the presence of additional cytochrome c/dATP. However, the inhibitory protein TUCAN does not play a role in inhibition of procaspase-9 and in determining the sensitivity to cisplatin in NSCLC.

## Background

Lung cancer is the major cancer killer and a health care problem worldwide with an overall 5-year survival rate of less than 15 %. Non-small cell lung cancer (NSCLC) represents 80% of all cases of lung cancer [1,2]. The cornerstone therapy for NSCLC is surgery, but this is radical in only about 30% of cases. Patients with a more advanced stage and radically operated patients are candidates for systemic chemotherapy, which has however a low level of efficiency.

Resistance to apoptosis in tumor cells can hamper the curative effect of chemotherapy, and several studies have demonstrated apoptosis resistance in NSCLC [3]. At the molecular level, the caspases are responsible for the execution of apoptosis [4,5], and the efficacy of caspase-activation in tumor cells in response to treatment will, at least in part, determine the therapeutic effect [6]. Two main caspase-dependent cell death pathways have been identified [7,8]. The intrinsic pathway is triggered upon disruption of the mitochondria, leading to the release of cytochrome c into the cytosol where it induces apoptosome formation and caspase-9 activation [9]. The extrinsic pathway, on the other hand, is initiated via death receptors on the cell membrane, such as tumor necrosis factor receptors. After ligand-induced trimerization, the receptors recruit the cytosolic death-domain-containing protein FADD (Fas-associated protein with death domain) to form the death-inducing signalling complex (DISC), which mediates the activation of procaspase-8 [8]. The initiator caspases, activated in the apoptosome (caspase-9) or DISC (caspase-8) can, in turn, cleave and activate the executioner caspases-3, -6 and -7, causing irreversible apoptosis.

The activation of caspases needs to be tightly regulated, and members of the inhibitor of apoptosis protein (IAPs) family are known to directly bind to and inhibit caspases through their baculovirus-IAP-repeat domain (BIR) [10]. In addition to the BIR domain, certain IAPs contain a caspase recruitment domain (CARD), which is also present in other apoptosis-related proteins, such as TUCAN (tumor-up-regulated CARD-containing antagonist of caspase-nine), also called CARDINAL or CARD8 [11,13,14]. TUCAN was reported to be involved in inhibition of apoptosis by interfering with Apaf-1 binding to procaspase-9 via its CARD domain [11]. Moreover, a novel isofrom of TUCAN has been recently reported to obstruct apoptosis headed by both caspase-8 and caspase-9 [12].

We, and others, have provided evidence that a blockade of the mitochondrial apoptotic pathway in NSCLC plays an important role in drug resistance [15-17], but the precise mechanism underlying this blockade is still unclear [18]. In this study, we have investigated a potential role of TUCAN as a caspase-9 inhibitor in NSCLC. Our results show that TUCAN is expressed at high level in NSCLC cells when compared to small cell lung cancer (SCLC) cells. However, no interaction between TUCAN and (pro)caspase-9 was detected, and RNA interference-mediated down-regulation of TUCAN did not restore cisplatin-induced caspase-9 activation or affect cisplatin sensitivity. We conclude that TUCAN is not responsible for inhibition of caspase-9 in NSCLC cells, and that its role in modulation of apoptosis is more complex than initially proposed.

## Methods

### Cell culture and drug treatment

Human NSCLC NCI- H460, -A549 and -H322 and SCLC NCI-GLC4, -N417 and -H187 cells were grown in RPMI medium (Cambrex Bioscence, Verviers, Belgium). NSCLC-SW1573 cells and the packaging cell line, PT 67, were cultured in DMEM (Cambrex Bioscence, Verviers, Belgium). Media were supplemented with 10% FCS, 100 units/ml penicillin, and 100 μg/ml streptomycin (Gibco BRL, Invitrogen Corp., Scotland, UK). Cells were plated 24 h before treatment and, when applicable, treated with cisplatin (Bristol-Myers Squibb, Woerden, Netherlands) or with etoposide (Sigma- Aldrich, Saint Louis, MI) for 24 or 48 h. In the experiments an IC80 concentration of cisplatin was used that represents a clinically relevant concentration.

### Plasmids and cloning

The plasmids encoding a MYC-tagged cleavage-resistant procaspase-9 mutant, TUCAN-flag, and Apaf-1 (1–570) were described before [19-21]. pcDNA3-171-431 FLAG-TUCAN, pcDNA3-341-431 FLAG-TUCAN and TUCAN-YFP were generated by PCR-based cloning strategies using TUCAN-flag as a template. The caspase-9 cDNA was obtained by RT-PCR from RNA isolated from H460 cells, and sequenced using an ABI 310 Capillary DNA Sequencer/Genotyper (Applied Biosystems, Forest City, CA). Sequence comparison was done using BLAST, and the published sequence of procaspase-9, [GenBank: XM_001584][22].

### RNA interference

The pSUPER and pSUPERretro vectors were obtained from R. Agami [23]. For the silencing of TUCAN three different 19-bp oligonucleotides were designed: 1) 5'-tcgtcagtttctggggcct-3; 2) 5'-tgtggatgttgagttgatt-3' and 3) 5'-agcgacgccttgctaacaa -3'. Oligonucleotides were cloned into Hind III/Bgl II- digested pSUPER. For stable transfection, the inserts from the pSUPER-TUCAN -1, -2, -3 vectors were subcloned into the EcoRI/XhoI sites of pSUPERretro, yielding pSUPERretro-TUCAN -1, -2, -3 constructs. Next, PT67 packaging cells were transiently transfected using FuGene (Roche Diagnostic, Mannheim, Germany) with the individual pSUPERretro plasmid to produce retroviral particles. For stable transfection, H460 cells were grown for 3 days and daily exposed for 6 h to fresh retroviral supernatant for optimal infection. Selection for stable transfectants was performed in medium containing 1, 0–1,5 μg/ml of puromycin for 1-1,5 week. As negative control, a pSUPERretro vector containing the TUCAN-1 insert in reverse orientation was used.

### Real Time RT-PCR

Total RNA was extracted from stable transfected H460 cells using RNAzol and reverse transcribed into cDNA prior to semi-quantitative RT-PCR analyses with a LightCycler (Roche Diagnostics GmbH Mannheim, Germany). The forward and reverse primers used for TUCAN were 5'-gcctttgtgaaggagaaccaccgg-3' and 5'-ctccaccatgctccgcaaggc -3', and for GAPDH 5'-accacagtccatgccatcac-3' and 5'-tccaccaccctgttgctgta-3'. Relative mRNA expression was calculated as: (E^-Cp target gene^)/(E^-Cp reference gene^), in which E = efficiency and Cp = crossing point. GAPDH was used as internal standard and reference gene. The efficiency (GAPDH, 1,77; TUCAN, 1,99) was calculated for all target and control genes using a cDNA concentration range.

### Apoptosis and caspase-9 activity assays

The extent of apoptosis was determined by flow cytometry, using either PI (Sigma) staining of hypodiploid DNA. The percentage of specific apoptosis was calculated by subtracting the percentage of spontaneous apoptosis of the relevant controls from the total percentage of apoptosis. Caspase-9 activity was assayed in cellular extracts using a caspase-9 activity kit (MBL Co., Nagoya, Japan) according to manufacturer's instructions. Fluorescence was detected using Spectra Fluor equipped with a 400-nm excitation and a 505-nm emission filter (Tecan, Salzburg, Austria). Fold-increase in the protease activity, as measured by LEHD-AFC cleavage, was determined by comparing the levels of treated cells with untreated controls [15].

### Western blotting

Western blotting was performed as described before [24]. The following primary antibodies were used: anti-caspase-9 rabbit polyclonal (Cell Signaling Technology, Beverly, MA), anti -TUCAN/CARDINAL rabbit polyclonal (kindly provided by Dr. J.C. Reed, Burnham Institute, La Jolla, California and Dr. S.J. Martin, The Smurfit Institute, Trinity College, Ireland), anti-Apaf-1 mAb (R&D System, Minneapolis, MN) and anti-β-actin mAb (Sigma- Aldrich, Saint Louis, MI). As secondary antibodies, horseradish peroxidase (HRP)-conjugated goat anti-mouse or anti-rabbit (Amersham, Braunschweig, Germany), were used.

### Protein interaction and *in vitro*-translation assays

Immunoprecipitation was performed as described by Hill *et al*. [25]. Confluent cell cultures were washed in ice-cold PBS, harvested, and resuspended with lysis buffer, consisting of 20 mM HEPES-KOH [pH 7.5], 10 mM KCl, 1.5 mM MgCl_2_, 1 mM EDTA, 1 mM EGTA, 1 mM dithiothreitol (DTT), and 1× Protease Inhibitor Cocktail (PIC, Roche Diagnostics GmbH, Mannheim, Germany), 250 μM PMSF and 1 mM Na_3_VO_4_. After incubation on ice and homogenization with 45 strokes with a Dounce homogenizer, the extracts were centrifuged for 30 min at 4°C. The protein concentrations were determined according to Bradford (Bio-Rad, Veenendaal, The Netherlands). Apoptosome formation was induced by incubation with 50 μg/ml cytochrome c (Sigma, Saint Louis, MI) and 1 mM dATP (Roche Diagnostics GmbH, Mannheim, Germany) for 45 min at 37°C. Prior to immunoprecipitation; the extracts were supplemented with 3- [(3-Cholamidopropyl) dimethylammonio]-1-propanesul-fonate (CHAPS) and NaCl. Extracts were incubated with anti-caspase-9 antibody (Upstate Biotechnology, Charlottesville, VA) overnight at 4°C. Immunocomplexes were pulled down with protein A/G sepharose beads (Santa Cruz, CA), and proteins were detected by Western blotting. Immunoprecipitation studies with *in vitro *translated radiolabeled proteins (TNT system, Promega, Madison, WI) were performed in lysis buffer, consisting 20 mM Tris [pH 8], 50 mM NaCl, 2 mM EDTA, 0,1%NP40, 1× Protease Inhibitor Cocktail (PIC), 250 μM PMSF and 1 mM Na_3_Vo_4_. For pull-down the caspase-9 (Upstate Cell Signaling Solutions, Charlottesville, VA) or the M2 anti-FLAG (Sigma, Aldrich, Saint Louis, MI) antibodies were used. Proteins were separated and visualized as described previously [26].

### Statistical analysis

When applicable results were analyzed by the Student's *t *test. All *P *values were considered significant when p ≤ 0.05. Statistical analysis was performed using the SPSS software program 9.0 (SPSS, Chicago, Ill).

## Results

### Analysis of components of the apoptosome and processing of caspase-9 induced by exogenous cytochrome c and dATP in lung cancer cells

**Figure 1:**
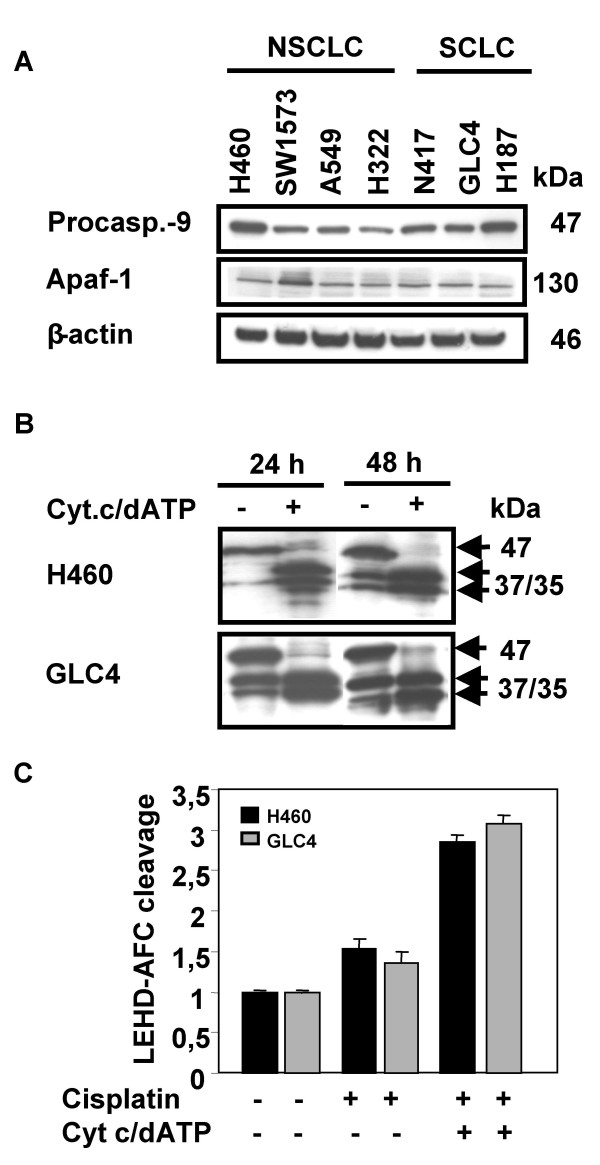
**Caspase-9 expression and processing in NSCLC and SCLC cell line**. A) Western blots showing the level of expression of procaspase-9 and Apaf-1 in a panel of NSCLC and SCLC cell lines. β-actin was used as control for protein loading. B) H460 and GLC4 cells were treated with IC80 concentrations of cisplatin for 24 and 48 h or C) only for 48 h and cellular extracts were generated. Extracts were incubated for 45 min at 37°C in the presence (+) or absence (-) of exogenously added cytochrome c and dATP. Subsequently, caspase-9 was immunoprecipitated and visualized by western blotting or activation of caspase-9 was measured by the cleavage of the fluorogenic substrate LEHD-AFC, respectively.

Given our previous finding that procaspase-9 is inhibited in NSCLC H460 cells [15], we started our investigation by examining the expression levels of the main apoptosome components Apaf-1 and caspase-9, in a broader panel of lung cancer cell lines, including the NSCLC cell lines H460, SW1573, A549 and H322, and the SCLC cell lines GLC4, N417 and H187 by Western blotting. In all cell lines, we observed a comparable level of caspase-9 and Apaf-1 proteins (Fig. 1A), indicating that altered expression of these proteins is unlikely to account for the lack of caspase-9 activation in NSCLC cells. In addition, we sequenced the ORF of caspase-9 amplified from NCI-H460 cells, which revealed no sequence alteration (not shown).

Next, we examined the processing of caspase-9 in two representative lung cancer cell lines, H460 (NSCLC) and GLC4 (SCLC), after treatment for 24 and 48 h with IC80 concentrations of cisplatin. Extracts were split in two and one half was supplemented with cytochrome c and dATP, whereas the other half remained untreated. Subsequently, the lysates were analysed for caspase-9 cleavage by immunoprecipitation using an anti-caspase-9 antibody and Western blotting. In the absence of exogenously added cytochrome c and dATP hardly any caspase-9 cleavage products of 37 and 35 kDa were detected in H460 extracts after 24 h cisplatin treatment; however, at 48 h post-treatment some cleavage was apparent (Fig. 1B). In GLC4 cells, we could observe cleavage of caspase-9 already after 24 h treatment. Thus, cleavage of procaspase-9 appears to occur faster in the SCLC cell line. Examination of caspase-9 activity by fluorescence-based assays showed a non-significant increase after cisplatin treatment in H460 (p = 0.06) and GLC4 cells (p = 0.069). Importantly, we noticed that addition of cytochrome c and dATP triggered a complete processing of procaspase-9 and resulted in a 2-3-fold increase in caspase-9 activity (Fig. 1B,C). Altogether, these observations indicate that inhibition of caspase-9 activation in lung cancer cells is not caused by an intrinsic defect in the apoptosome, and suggested to us the presence of (a) inhibitory protein(s) that block(s) the activation of capsase-9

### TUCAN is highly expressed in NSCLC cells

**Figure 2:**
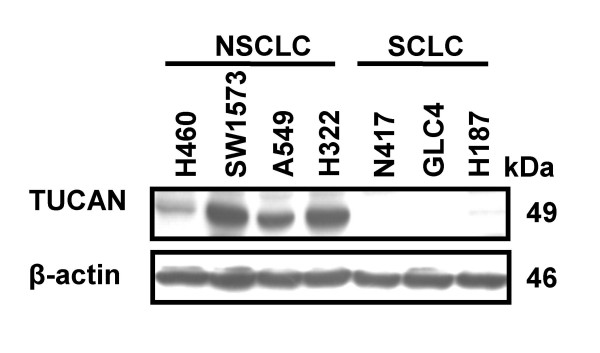
**TUCAN expression in a panel of NSCLC and SCLC cells**. The expression of TUCAN was determined in the indicated panel of cells by Western blotting. β-actin was used as control for protein loading.

As a possible candidate for caspase-9 inhibition we tested the expression of the reported caspase-9 inhibitor of Apaf-1-mediated caspase-9 activation, TUCAN [11], in the panel of NSCLC and SCLC cells (Fig. 2). Interestingly, a high expression level of TUCAN was observed in NSCLC cells, whereas this protein was hardly detectable in SCLC. Thus, we considered TUCAN as a promising candidate for caspase-9 inhibition in NSCLC.

### No evidence for TUCAN – procaspase-9 interaction

**Figure 3:**
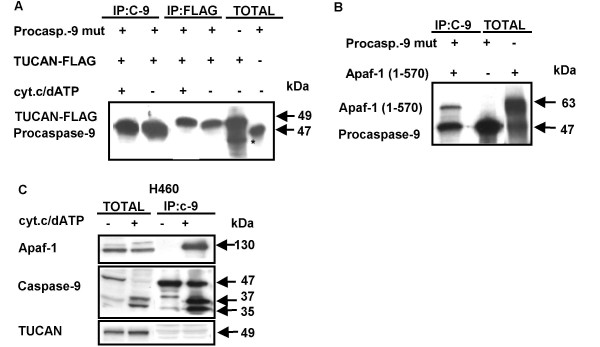
**TUCAN does not interact with procaspase-9 or Apaf-1**. Immunoprecipitation of *in vitro *translated procaspase-9 (procaspase-9 mut) mixed with a FLAG-tagged version of TUCAN (TUCAN-FLAG) (A), and procaspase-9 mixed with a truncated variant of Apaf-1 (Apaf-1 (1–570)) as positive control (B). Immunoprecipitation was performed with anti-caspase-9- or anti-Flag antibodies. The total of radiolabelled proteins in the *in vitro*-translation mixture prior to immunoprecipitation is also included (total). A low molecular weight band obtained in the total TUCAN mixture that did not react with the anti-Flag antibody is indicated with an asterisk. C) H460 cell extract was subjected to caspase-9 pull-down in the presence (+) or absence (-) of exogenously supplied cytochrome c and dATP followed by western blot detection of caspase-9 and TUCAN, or Apaf-1 as positive control. As a control, total cell extract (total) was included.

In order to further evaluate a potential role for TUCAN in inhibiting caspase-9 activation in NSCLC cells, we first tested the interaction between procaspase-9 and FLAG-tagged TUCAN by immunoprecipitation assays using *in vitro *translated ^35^S-methionine-labelled proteins. As shown in Fig. 3A, pull-down experiments with either TUCAN-flag or caspase-9 specific antibodies could not demonstrate a direct interaction between these proteins. The addition of cytochrome c and dATP prior to pull-down did not alter the outcome of the experiment. As positive control, the interaction between *in vitro*-translated, non-cleavable procaspase-9 and the amino-terminal CARD-containing part of Apaf-1 (Apaf1 (1–570)) was readily detectable under these conditions (Fig. 3B). To exclude the possibility that the carboxyl-terminal CARD domain of TUCAN was masked in some way by its amino-terminal part we generated two deletion mutants, named FLAG-TUCAN (171–431) and FLAG-TUCAN (341–431). However, no interactions between these TUCAN deletion mutants and procaspase-9 were detected, either in the presence or absence of cytochrome c and dATP (data not shown). These observations indicate that the CARD domain of Apaf-1, but not the CARD domain of TUCAN interacts with procaspase-9. These results are in line with earlier observations by Bouchier-Hayes *et al*. [14] who also could not demonstrate direct interactions between caspase-9 and TUCAN.

To examine the possibility that cellular cofactors or posttranslational modifications of the proteins are required for CARD-domain-mediated TUCAN/caspase-9 interactions, we also studied interactions between endogenous caspase-9 and TUCAN. As a positive control, we determined the interaction between endogenous procaspase-9 and Apaf-1. Consistent with the notion that the association of Apaf-1 and procaspase-9 might be suppressed in H460 cells, Apaf-1/caspase-9 interaction was detected only in the presence of exogenously added cytochrome c and dATP (Fig. 3C). Under the same conditions, we could not detect an interaction between TUCAN and procaspase-9 neither in H460 cells nor in SW1573 cells (not shown) that express higher levels of TUCAN (see Fig. 2).

### Down-regulation of TUCAN has no effect on cisplatin-induced apoptosis

**Figure 4:**
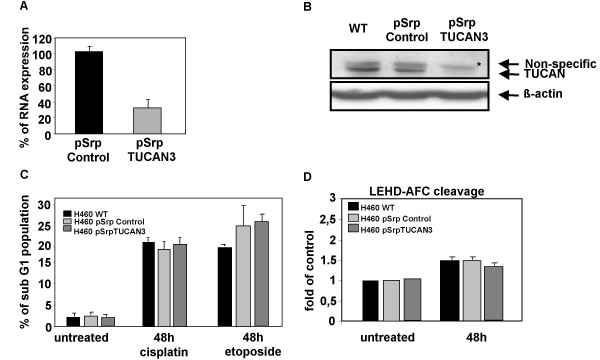
**Effect of TUCAN down-regulation on cisplatin sensitivity in H460 cells**. **A) **Real time RT- PCR experiment and B) Western blot analysis showing the silencing of TUCAN expression in H460 cells stably transfected with pSUPERretro TUCAN3 (pSrpTUCAN3). As control, a construct containing the reverse orientation of the RNA interference sequence, pSUPERretroTUCAN-control (pSrp control), was used. The mean ± SD of three independent experiments is shown, * – non-specific band. **C) **Cisplatin- and etoposide-induced apoptosis in H460 cells and derived transfectants. The percentage of cells in the sub-G_1 _fraction was taken as the apoptotic population. The results are depicted as the means of three experiments; *bars*, SD.**D) **activation of caspase-9 was measured by the cleavage of the fluorogenic substrate LEHD-AFC in H460 cells and derived transfectants exposed to cisplatin.

The potential influence of TUCAN on procaspase-9 cleavage and chemotherapy sensitivity was also investigated using an RNA interference approach. We designed three different TUCAN specific RNA-interfering oligonucleotides that were cloned into the pSUPERretro vector (see material and methods). After the generation of stably transfected derivatives of H460, the silencing effect of each construct was tested by semi quantitative RT-PCR. The most effective TUCAN-silencing plasmid, pSUPERretro TUCAN3, resulted in an approximately 65% of transcript reduction (Fig. 4A; p = 0.000), and almost complete downregulation at the protein level as shown in Figure 4B. However, there was no significant increase in apoptosis observed in cisplatin-exposed H460 cells with downregulated TUCAN expression and parental H460 cells (p = 0.808) or H460 cells with the control vector (p = 0.77). As an additional test also no significant differences in apoptotic cell death was observed in this cell panel after treatment with etoposide (p = 0.054; p = 0.768, respectively) (Figure 4C). Furthermore, the silencing of TUCAN did not show a significant change in cisplatin-induced caspase-9 activity as measured by fluorescence-based assays, neither when compared to parental H460 cells (p = 0.808) nor control vector containing ones (p = 1.00). Altogether, the results of the immunoprecipitation and RNAi experiments strongly suggest that TUCAN does not play a role as caspase-9 inhibitor in NSCLC.

## Discussion

In this study, we have investigated the potential role of TUCAN as a determinant of caspase-9 inhibition and resistance to chemotherapy in NSCLC cells.

We demonstrated that inhibition of caspase-9 in lung cancer cells could not be attributed to abnormal expression of Apaf-1 and procaspase-9 or to genetic alterations in the procaspase-9 coding sequence analysed in NSCLC H460 cells. In fact, exogenous supplementation of cytochrome c and dATP in cell extracts stimulated the cleavage and activation of procaspase-9 in lung cancer cells, suggesting a presence of an inhibitor(s), whose effect may be overcome by an excess of cytochrome c/dATP. Interestingly, the cleavage of procaspase-9 in cell extract treated only with cisplatin appeared earlier in GLC4 than in H460 cells, suggesting that the regulation of this pathway may be different in particular lung cancer subtype. This is consistent with previous data showing that γ-irradiation-induced cleavage of procaspase-9 was delayed in NSCLC cells, when compared with SCLC cells [28]. This previous report also showed much more appreciable activation of caspase-9 in lung cancer cells after treatment with γ-irradiation than with the cytotoxic drug etoposide. Similarly, in our study, we detected no significant increases in caspase-9 activity in H460 and GLC4 cells after cisplatin treatment. Since these observations suggest the presence of an inhibitor of caspase-9 activity in lung cancer cells, and based on the reported binding of TUCAN to procaspase-9 via their CARD motifs leading to caspase-9 inactivation [11], a possible role of TUCAN in blocking caspase-9-mediated apoptosis in lung cancer cells was explored. In Western blotting experiments, a strong expression of TUCAN was observed in a panel of NSCLC cell lines when compared to SCLC cells. Next, we explored the role of TUCAN in inhibition of procaspase-9 in NSCLC cells. In co-immunoprecipitation experiments with both *in vitro*-translated proteins and cell extracts, we could not obtain evidence for TUCAN binding to (pro)caspase-9. In this context, it should be noted that conflicting results have been reported on this interaction, which may be related to experimental and cell specific differences [11,14]. Under our experimental conditions, TUCAN does not seem to interact with caspase-9. In line with these results, down-regulation of TUCAN by RNA interference did not restore procaspase-9 processing, and did not influence cisplatin sensitivity in NSCLC cells. Altogether, our results do not support a role for TUCAN as inhibitor of caspase-9 in NSCLC cells.

Although, TUCAN does not seem to act as a caspase-9 inhibitor, its differential expression in NSCLC versus SCLC cells suggests that TUCAN may play a role in the lung cancer biology, specifically in the NSCLC subtype. In this regard to their role in apoptosis, several CARD-containing proteins have been implicated in signalling pathways that modulate the NF-kappaB transcription factor, which regulates cell survival and may serve as therapeutic targets in cancer [29,30]. In the case of TUCAN, CARD-dependent interactions have been reported not only with caspase-9, but also with procaspase-1, and with the p53-responsive gene DRAL [13,20]. Moreover, a CARD-independent interaction of TUCAN with IKKγ (NEMO) has been described, resulting in the inhibition of interleukin-1 and TNF-induced NF-κB activation [14]. The function of TUCAN and CARD proteins in general appears to be more complex than initially assumed. Further studies are required to unravel the role of TUCAN in tumor biology as well as to elucidate the molecular basis of caspase-9 inhibition in lung cancer cells.

## Conclusion

These results indicate that procaspase-9 is functional and can undergo full processing in the presence of additional cytochrome c/dATP. However, the inhibitory protein TUCAN detectable only in NSCLC cells does not play a role in inhibition of procaspase-9 and in the determining of sensitivity to cisplatin.

## Competing interests

The author(s) declare that they have no competing interests.

## Authors' contributions

AC: designed and performed experiments, analysed data, wrote manuscript

GG: project leader, analysed data, wrote manuscript

BSJH: performed experiments, analysed data

CGF: analyzed data

JAR: wrote manuscript

FAEK: project leader, designed experiments, analysed data, wrote manuscript

## Pre-publication history

The pre-publication history for this paper can be accessed here:
